# A Preliminary Study in Er:YAG Laser Debonding of Lithium Disilicate Crowns: Laser Power Setting vs Crown Thickness

**DOI:** 10.7150/ijms.85722

**Published:** 2023-08-06

**Authors:** Rifat Gozneli, Tansu Sendurur, Esra Silahtar

**Affiliations:** 1 Associate Professor, Department of Prosthodontics, Faculty of Dentistry, Marmara University, Istanbul, Turkey.; 2 Research Assistant, Department of Prosthodontics, Faculty of Dentistry, Marmara University, Istanbul, Turkey.; 3 Prosthodontist, Private Clinic, Istanbul Turkey.

**Keywords:** Crown removal, Debonding, Er:YAG laser, Heat transmission, Lithium disilicate

## Abstract

**Background:** Er:YAG laser is widely used in debonding of all-ceramic veneers nowadays. However, the data about laser parameters in all-ceramic crown removal is limited. The aim of this preliminary study was to determine the most appropriate laser parameters at safe heat transmission values ​​for lithium disilicate crowns in different thicknesses.

**Methods:** Twenty-seven intact premolars were prepared to fabricate lithium disilicate CAD/CAM full-coverage crowns in three different thicknesses: 1, 1.5 mm, and mixed thickness (n=9). Each thickness group was divided into 3 subgroups and subjected to Er:YAG laser at different wattages (5, 5.6 and 5.9 W) to determine the appropriate wattage for each thickness. The removal time and temperature rise values were recorded. The Kruskal-Wallis test was performed to evaluate any significant differences in removal time, Mann-Whitney U test with Bonferroni correction for multiple comparisons, and the Pearson chi-square test for temperature rise over the critical value (*p*<0.05).

**Results:** Laser irradiation at 5 W was safe and efficient for 1 mm thickness, while not efficient for others. Laser application at 5.9 W was efficient for all thicknesses, but, not safe for 1 mm thickness. The statistically significant difference in removal time was only between 5 W and 5.9 W groups (*p*=0.035). Pearson's chi-square test revealed that the temperature rise after 5.9 W laser application was significantly different from 5 W in 1 mm thickness group (*p*=0.043).

**Conclusion:** Er:YAG laser lithium disilicate crown removal is an effective and safe method using laser settings appropriate for crown thickness.

## Introduction

In the last 20 years, aesthetic ceramic materials have become very popular in the aesthetic zone 1,2. The survival of an all-ceramic restoration depends on the strength and durability of the adhesion complex between three components: the ceramic material, resin cement, and tooth surface. There is a high bond strength between these 3 structures 3. However, these restorations have to be removed when there are problems such as: marginal failure, improper placement of the restoration during cementation, ceramic fracture, possible endodontic therapy, discoloration or any aesthetic reasons 3,4. The conventional crown removal methods made with trial crown tractors, chisels, manual or automatic removers, and burs cause discomfort and pain 5. In addition, the bur may reach deep into the tooth structure accidentally when grinding since the color of all 3 structures is relatively white and visual differentiation is difficult to obtain 6. A successful crown removal relies on preserving the tooth structure from an iatrogenic damage 7. The lasers were introduced to prevent damage as a comfortable, safe, and conservative all-ceramic removal method 8. Another advantage of laser debonding is that the removed all-ceramic restoration can be rebonded, preventing to waste of time and money for the patient and the dentist 9. Different laser types were used for this purpose, such as; carbon-dioxide (CO_2_) 10, neodymium-doped yttrium aluminum garnet (Nd:YAG) 11, diode 12, ytterbium fiber 13, and erbium-doped yttrium aluminum garnet (Er:YAG) 14. Laser debonding is provided by the degradation of resin cement via different mechanisms, such as thermal softening, thermal ablation, and photoablation 11. Thermal softening causes a large temperature rise in tooth structures since it is a relatively slow method, and occurs when CO2 or Nd:YAG lasers are used 9. The most effective and safe laser type for removal of all-ceramic restorations is Er:YAG laser (2940 nm) due to its high absorption capability in dental hard tissues and resin cements, which contain H2O molecules 5. Thermal ablation heats up the resin instantly and the resin vaporized rapidly. The OH ions in resin cement absorbs the transmitted energy and ablation occurs 15. When sufficient resin is ablated, bond strength decreases and the restoration is easily separated from the tooth surface 11. Photoablation occurs with a high laser energy, which enhances the energy levels of the bond between the resin atoms above their dissociation levels. Er:YAG laser debonding is mostly based on thermal ablation and photoablation mechanisms 15.

The dangerous aspect of laser light to be aware of is the heat created in the targeted tissue. In order not to endanger the vitality of the tooth, the heat transmission towards the pulp should be controlled. Irreversible changes may occur in the pulp as a result of thermal effects. The minimal 5.5 °C temperature increase may cause loss of tooth vitality 16. Necrotic changes are observed in the dental pulp after 16.7 °C temperature increase 17. It is important not to exceed these critical values during laser debonding.

There is a considerable number of studies about the bracelet and laminate veneer debonding with Er:YAG laser 18-21, whereas, the studies on full-coverage all-ceramic crown debonding and the proper laser parameters are limited 7,17,22. Therefore, the aim of this pilot study was to determine the most appropriate laser parameters for the laser removal of lithium disilicate crowns in different thicknesses, at safe heat transmission values​​. The null hypothesis for this study was that high laser energies would be necessary in thick crowns, but, high energies would cause damage in thinner ones. By the results of this study, the laser parameters appropriate for the removal of crowns in each thickness and safe for vital tissues underneath; will be used in extended future studies on all-ceramic crown debonding.

## Materials and Methods

Different scenarios were constructed to test the different laser parameters in terms of safe heat transmission values during Er:YAG laser debonding of lithium disilicate crowns in different thicknesses. Twenty-seven intact human premolars, extracted for periodontal and orthodontic reasons were decontaminated with 0.1% thymol solution for 24 h and stored in distilled water until use. The teeth were embedded in C-type silicone blocks (Zetaplus Putty; Zhermark SpA, Badia Polesine, Italy) and tooth reduction was performed with a chamfer finish line using a conical-shaped chamfer bur (Ökodent Gruppe, Tautenhain, Thüringen, Germany). The reductions were made with a high-speed handpiece fixed on a surveyor (KaVo EWL Typ 990; Kavo Elektrotechnisches Werk GmbH, Leutkirch im Allgau, Germany). The prepared tooth surfaces were scanned using an inEos X5 device (Dentsply Sirona Charlotte, NC 28277, USA). After scanning, the crowns were designed as maxillary first premolar in three different thicknesses: 1 mm, 1.5 mm, and mixed thickness (marginal 1/3: 1 mm, middle 1/3: 1.5 mm and occlusal 1/3: 2 mm) (n=9), with inLab 18.1 program (Dentsply Sirona Charlotte, NC 28277, USA). The IPS E.max CAD blocks (MT, A1; Ivoclar Vivadent, Schaan, Liechtenstein) were milled using inLab MC X5 fabrication device (Dentsply Sirona Charlotte, NC 28277, USA) to fabricate the crowns. After the try-in of unsintered lithium disilicate crowns on the relevant teeth, the crystallization process was performed in a firing furnace (Programat P310; Ivoclar Vivadent, Liechtenstein). Each 9 samples were then divided into 3 subgroups to apply Er:YAG laser at three different wattages (5, 5.6 and 5.9 W).

After the crowns were fabricated, the cementation process was started. The prepared tooth surfaces were etched with 37% orthophosphoric acid gel (Total Etch; Ivoclar Vivadent, Liechtenstein) for 15 s, and then; washed and dried. The universal adhesive (Adhese Universal; Ivoclar Vivadent, Liechtenstein) was homogeneously applied to the etched surface with a brush for 20 s, diluted for 20 s with an air spray and light-cured for 10 s, as instructed by the manufacturer. The inner surfaces of the lithium disilicate crowns were acid etched with 4% hydrofluoric acid gel (IPS Ceramic Etching Gel; Ivoclar Vivadent, Liechtenstein) for 20 s, and then; washed and dried for 30 s. Silane (Monobond S; Ivoclar Vivadent, Liechtenstein) was applied to the etched ceramic surfaces for 60 s and each crown was cemented with a dual-cure resin cement (Variolink Esthetic DC; Ivoclar Vivadent, Liechtenstein). After the excess cement was removed, the samples were light-cured for 20 s at each surface.

**Figure 1:**
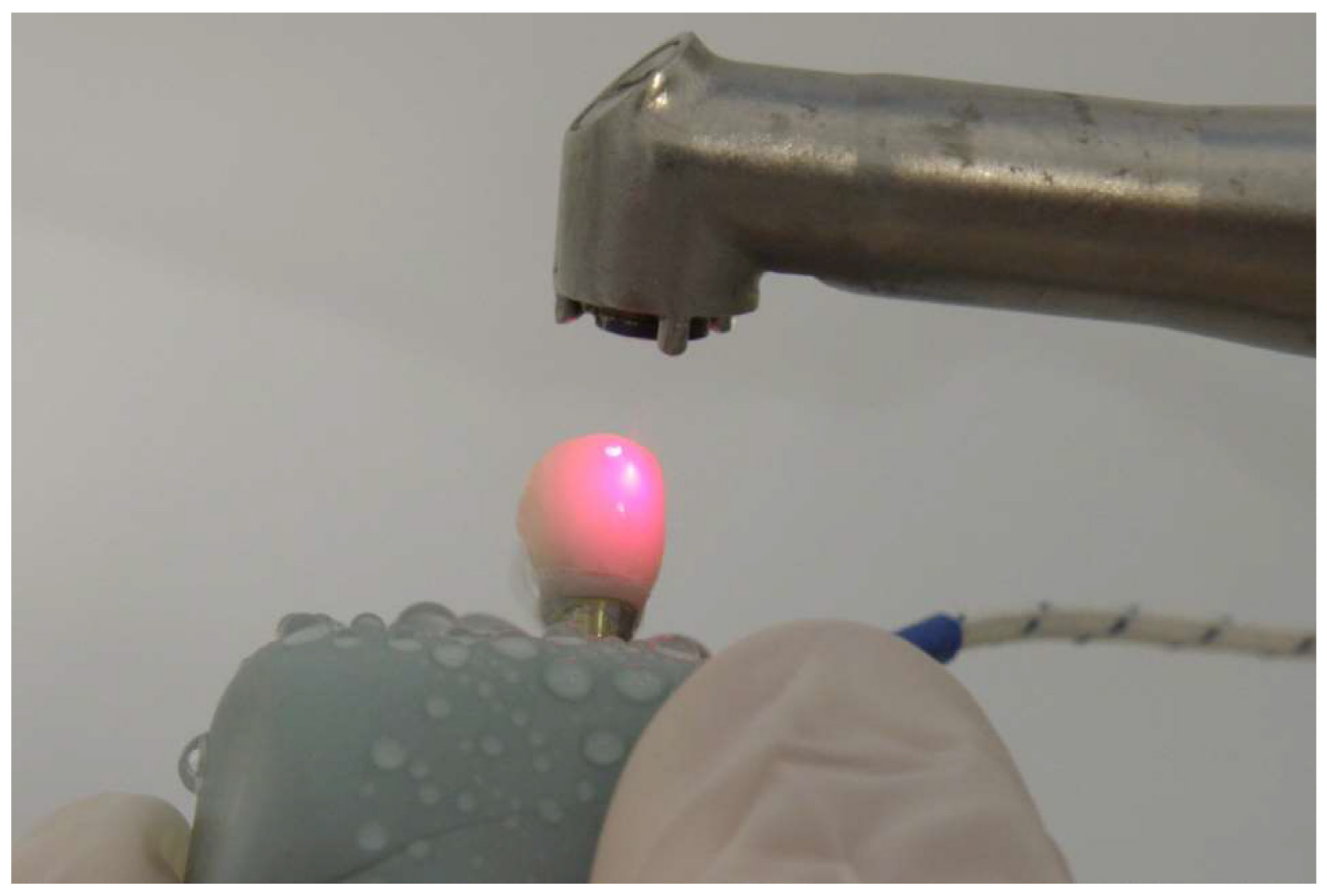
Er:YAG laser application for crown removal.

**Figure 2:**
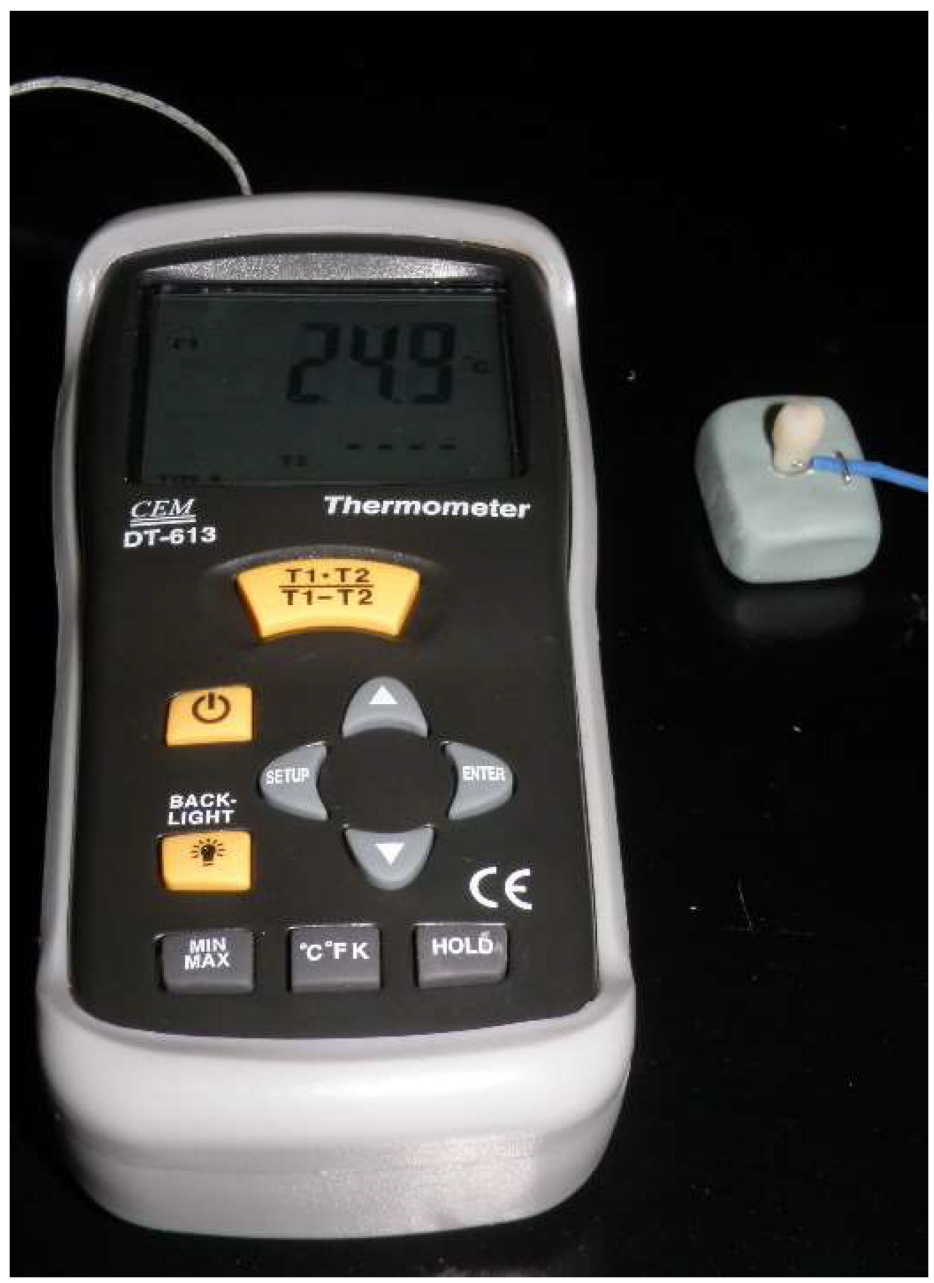
Micro-thermocouple testing device for measuring the heat transmission values during laser application.

The samples were kept in 37 °C distilled water in the incubator for 24 h. After 24 h, a solid-state Er:YAG laser (Fidelis III; Fotona d.o.o, Ljubljana, Slovenia) was used to remove the crowns (Fig. 1). Heat transmission values were measured in the pulp chamber during laser application using a micro-thermocouple testing device (Fig. 2). The maximum, minimum, and average temperature values during the laser application period were automatically recorded on the micro-thermocouple device.

### Laser Parameters and Application

The laser properties and parameters tested in the study are listed in Table 1.

The laser application was performed by scanning method in a zigzag pattern at a distance of 7-8-mm to the ceramic surface, initially at the buccal surface. The laser was applied up and down from the incisal margins to the cervical margins for 30 s. The same application pattern was repeated on the palatal surface for 30 s. After buccal and palatal surfaces, the laser was applied to the buccal and palatal line angles/cusps for 30 s; 15 s for buccal line angles/cusps, and 15 s for palatal line angles/cusps. Irradiation was then applied to the occlusal surface, mesially to distally for 30 s. Finally, the laser was applied to the interproximal areas from both; the lingual and buccal sides for 30 s. The total laser application period was 2 min 30 s for the first laser application. After the first application, crown removal was attempted. When no movement was detected the laser application was repeated at half of the first duration at each application surface and removal was attempted again. Attempts at crown removal were made using a Heidemann spatula, and the crown was scratched off from the gingival margin. The total laser application period was limited to 15-minutes for each sample.

**Figure 3:**
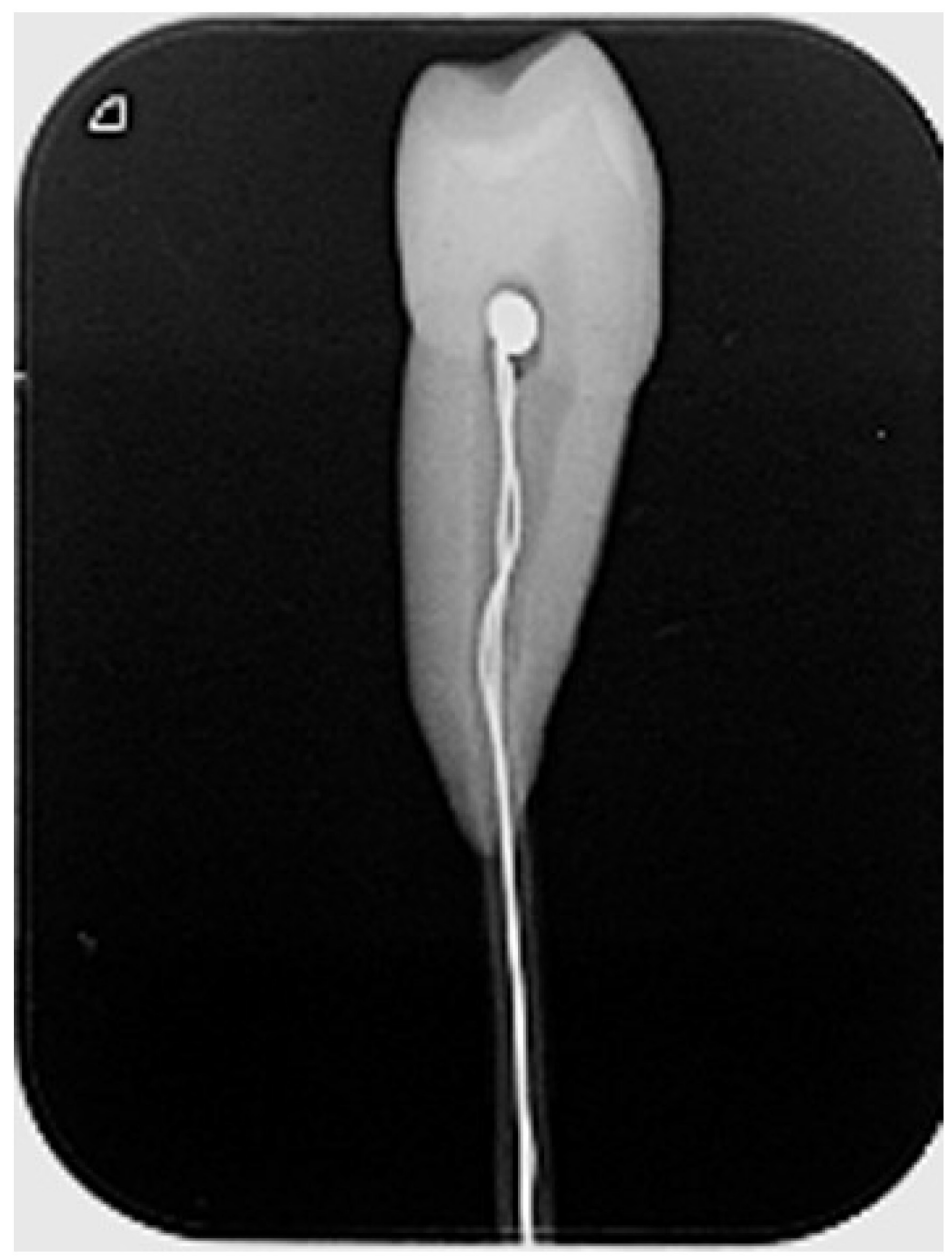
Micro-thermocouple type K probe placed in the pulp chamber.

To measure the temperature changes in the pulp chamber, a hole was drilled at the root surface using a round bur, and the micro-thermocouple tip was placed in the pulp chamber. Radiographs were used to confirm the correct tip position (Fig. 3).

One crown sample from each thickness group, which was removed both in the shortest time period and in safety temperature change, was examined using energy-dispersive X-ray spectroscopy (EDX) incorporated scanning electron microscopy (SEM) (ZEISS EVO MA10; Carl Zeiss Microscopy GmbH, Jena, Germany) at a magnification of x500. The structural integrity and surface of the laser irradiated crowns in different thicknesses were evaluated. EDX spectra were collected from the occlusal one-third of each sample that were different in thickness.

In addition to removal time, the highest temperature change values during laser application were noted for each sample. Statistical calculations were performed with the IBM SPSS V23 package program (Chicago, IL, USA). The Kruskal-Wallis test was used to evaluate the removal time periods in different laser parameters for each thickness. Mann-Whitney U test with Bonferroni correction was used for multiple comparisons. The Pearson chi-square test was used to examine the temperature changes over the critical value for different laser parameters. The statistical analyses were performed at a significance level of 0.05.

## Results

Resin-bonded lithium disilicate crowns, which were 1 mm in thickness were successfully laser-debonded and removed in one piece with all tested Er:YAG laser parameters. The median removal time obtained for 5 W was 225 s, 180 s for 5.6 W, and 165 s for 5.9 W. The statistically significant difference was only between 5 W and 5.9 W groups (p=0.035) (Table 2). While the critical temperature value was not exceeded (between 3.6-3.9 °C) with 5 W for samples 1 mm in thickness; it was exceeded in 66.7% (between 4.9-5.7 °C) of the samples irradiated with 5.6 W and 100% (between 5.9-6.3 °C) of samples irradiated with 5.9 W. Although, 5.9 W laser power provided shorter removal time values than 5 W, all samples' temperature rise values in 5.9 W group exceeded the critical value of 5.5 °C. Pearson's chi-square test revealed that there was a significant difference between 5 and 5.9 W laser application parameters related to the dental pulp safety in 1 mm thickness group (p=0.043) (Table 3). Thus, it would be more beneficial to use laser power of 5 W for lithium disilicate crowns 1 mm in thickness.

The samples 1.5 mm in thickness and mixed thickness were successfully laser-debonded and removed using 5.9 W. The median removal time was 375 s for 1.5 mm samples and 525 s for mixed thickness samples. Samples irradiated with the laser at 5 W and 5.6 W resulted unsuccessfully in removal even after 15 min (900 s) laser application. The use of 5.9 W was both effective and safe for 1.5 mm and mixed thickness groups since the temperature changes did not exceed the critical value of 5.5 °C.

Considering 5.9 W, the common laser parameter that provides success in debonding for all thickness groups, the median value for 1 mm group was 165 s, 375 s for 1.5 mm and 525 s for mixed thickness group. There was a significant difference between 1 mm and mixed thickness groups in removal time (p=0.021). The 1 mm thickness group had significantly shorter removal time than mixed thickness group. However, when the critical temperature change was taken into consideration for 1 mm, the temperature rise exceeded the critical value in 5.9 W laser application group. A short removal time will not be beneficial when the temperature rise is over the critical value.

**Figure 4:**
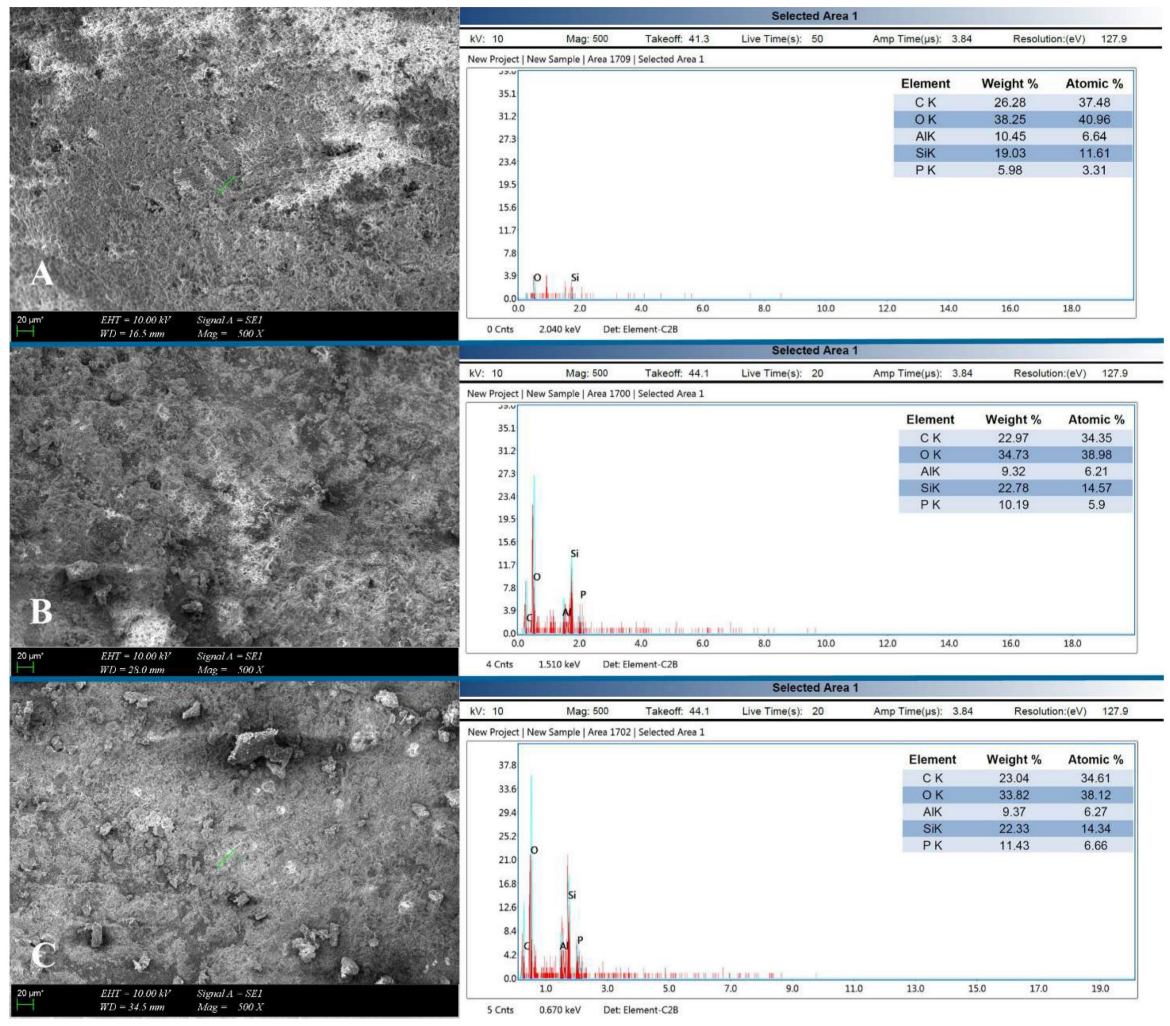
SEM images (x500) and EDX analysis of the laser irradiated and removed lithium disilicate crowns. (A) 1 mm thickness; 5 W laser power. (B) 1.5 mm thickness; 5.9 W laser power. (C) Mixed thickness; 5.9 W laser power.

SEM analysis was performed to examine the damage of the lithium disilicate crown surface. Examination showed that there were no cracks or fractures in macro or microstructure of the tested ceramic samples, which were in different thicknesses, related with the photoablation or thermal ablation. In the SEM images, ablated and carbonized resin cement remnants were observed (Fig. 4). The EDX area scans showed the percentages of C, O, Al, Si, and P elements. The elemental composition of the laser-debonded crowns in different thicknesses obtained by EDX analysis is listed in Fig. 4.

## Discussion

Although a considerable number of studies on laminate veneer debonding with Er:YAG lasers have been published 18-21, there are a limited number of studies on full-coverage all-ceramic crown debonding and removal 7,17,22. Except for clinical reports and non-evidence-based trials, the appropriate laser application parameters for Er:YAG laser debonding of a full-coverage all-ceramic restoration in different thicknesses luted on premolars have not yet been documented. This in-vitro pilot study, which was a preview of a prospective expanded study and aimed to determine the most appropriate laser parameters at safe heat transmission values ​​for lithium disilicate crowns in different thicknesses, has demonstrated that Er:YAG laser debonding is a safe and effective method for removal of resin-bonded all-ceramic crowns; when used with the appropriate parameters. Based on the results, the null hypothesis that high laser energies would be necessary in thick crowns, but, high energies would cause damage in thinner ones, was accepted.

One factor that can affect the debonding efficiency of laser irradiation on veneers is thickness. In the study of Cifuentes et al. 19, it was concluded that laser irradiation influenced the debonding of veneers in different thicknesses: easier debonding was observed in thinner veneers. Debonding of an all-ceramic crown was examined in two studies of Rechmann et al. 7,17, in which the crown samples were in standard thickness of 1 to 2 mm: 1.5 mm at contact points, 1.5 mm at non-functional cusps, 2.0 mm at functional cusps and 1 mm at margins. Gurney et al. 22, studied laser debonding using lithium disilicate discs, 1.5 mm in thickness with different laser parameters to determine the optimum parameter for debonding. However, the thickness of full-coverage all-ceramic restorations can range from 1 to 2 mm, and the application of a laser to debond crowns in different thicknesses has not been reported yet. In the current study, lithium disilicate crowns with thicknesses of 1 mm, 1.5 mm, and mixed thicknesses were tested to determine the optimal laser irradiation power for different thicknesses.

The tested parameters in the current study were designed by the reference of previous studies, which aimed to establish an ideal criterion for using the Er:YAG laser in debonding laminate veneers and full-coverage restorations 3,7,22. It was reported that a 2940 nm Er:YAG laser irradiation at a power of 5 W (100 mJ x 50 Hz) for 9 seconds provided the most effective debonding of lithium disilicate discs with a thickness of 0.7 mm and a diameter of 5 mm 3. Gurney et al. 22 stated that the lithium disilicate disc specimens irradiated with the laser output at 5 W allowed removal after the shortest exposure of laser cycles. Rechmann et al. 17 reported that the lithium disilicate full contour crowns on stand-alone teeth were removed using 5 W laser power (500 mJ x 10 Hz) and lithium disilicate crowns in an artificial row of teeth were removed using laser power of 5.6 W (varied between 5 and 5.9 W) at a pulse repetition rate of 10 Hz. In another study of Rechmann et al. 7, the lithium disilicate crowns on single, stand-alone molars were successfully laser debonded and removed with the laser parameter of 10 Hz pulse repetition rate and 560 mJ (5.6 W) varied between 500 and 590 mJ laser energy per pulse. In the present study, the tested laser parameters were 500, 560 and 590 mJ per pulse with a 10 Hz pulse repetition rate (5, 5.6, 5.9 W).

According to Albalkhi et al. 20 the debonding time and temperature change were affected by the pulse duration (PD) and water/air (W/A) cooling ratio. It was reported that laminate veneers were successfully debonded when the pulse duration was SSP (50 µs) or MSP (100 µs), which are efficient, fast, and safe parameters for the removal of lithium disilicate veneers. The W/A cooling ratio of 3/3 showed significantly the longest debonding time, while, 1/1 showed the highest elevation of pulp temperature with the same PD type 19. In the present study, PD of 100 µs with a 2/2 cooling ratio was used to keep tooth in safe.

Zhang et al. 21 stated that the laser tip was moved in scanning mode on the entire surface of the laminate veneers, both horizontally; and vertically. Rechmann et al. 7 started laser application from the occlusal surface, followed by moving over onto the line angles of the cusps, and then irradiating the buccal, lingual, and proximal surfaces. In the current study, laser application was started from the buccal surface, followed by palatal and proximal surfaces. Finally, the laser was applied to the line angles, cusps, and occlusal surface. The reason why it was initially applied to axial and proximal surfaces instead of to the occlusal surface was that the ablation at the occlusal surface could cause a ceramic fracture at the occlusal area because of the strict retention at the other surfaces.

There are a limited number of studies regarding the removal time of full-coverage all-ceramic crown removal with an Er:YAG laser. In the study of Rechmann et al. 7, it was defined that the range of irradiation time for debonding lithium disilicate crowns in mixed thickness varied from 85 to 420 s at pulse energies between 500 and 590 mJ with a 10 Hz repetition rate. In another study of Rechmann et al. 17, the debonding time of the samples was between 85 and 210 seconds where the laser parameter was fixed at 560 mJ pulse energy and a 10 Hz repetition rate. Both studies were performed using extracted human molars. In the current study, lithium disilicate crowns in different thicknesses were removed over longer time periods; since the crown samples were premolars and had higher mechanical retention properties related to their higher crown-height/crown-width ratio than molars.

Physical changes in the crown material may not be perceived visually after laser irradiation in crown removal procedure, thus causing a doubt in reusing the crown. Zhang et al. 23 reported that although no visible cracks were observed in any sample, there were microcracks in 5 W laser irradiated group in SEM images. In the current study, the SEM analysis of the lithium disilicate crowns subjected to Er:YAG laser for crown removal showed that the samples remained intact, without structural damage or any microcracks, similar to some previous reports 24-27.

This pilot study determining the appropriate laser parameter for lithium disilicate crowns in different thicknesses, has some limitations. Although, it was reported that a pilot study should have sample size of at least twenty participants 28, the larger sample size may affect the result and statistical significance. The height/width ratio of the prepared teeth may affect the debonding time of the laser-irradiated samples, although similar dimensions were tried to be obtained after tooth reduction. There are some limitations related with the potential differences between invitro conditions and the oral environment, such as; the lack of tooth vitality, blood circulation and saliva that would alter the temperature values; the application type of mechanical forces for removal and tooth location that would affect the debonding time and clinical result. Future experiments incorporating clinical simulation may alter the results. Another limitation of this study was that the resin bonded lithium disilicate crowns had no proximal contact with another tooth mesially and distally during laser irradiation. If the tested samples were in an artificial row of teeth, the removal time might be affected in the direction of increase. An additional limitation of the study was that the tested samples were premolars, which have different pulp chamber size than other type of teeth. The thermal conductivity might be changed in different types of teeth related with the pulp chamber size.

## Conclusions

Based on the results and within the limitations of this study, the following conclusions were drawn:

1. Lithium disilicate crown removal using Er:YAG laser is an effective and safe method, when performed with the laser parameter appropriate for crown thickness.

2. It would be safe not to increase the laser power over 5 W for lithium disilicate crowns 1 mm in thickness. Preventing tooth damage is more important than a short removal time.

3. The laser power of 5.9 W was both effective and safe for crowns in 1.5 mm and mixed thicknesses.

4. From the clinical aspect of view, when the thickness is known, the appropriate parameter provides success. However, when not known, it would be safer to apply Er:YAG laser at a power of 5 W first, and, then increase it, or, laser application to cervical areas at a power of 5 W, to middle at 5.6 W and to occlusal surfaces at 5.9 W.

Thanks to the results of this pilot study, the laser parameters determined for different thicknesses will be used in extended future studies as a standard parameter.

## Figures and Tables

**Table 1:** The laser properties and parameters tested in the study.

Er:YAG Laser Application	
Laser type	Solid-state
Wavelength	2940 nm
Delivery system	7-mirror articulated arm
Water/Air cooling	2/2 (14 ml/min)
Hand piece type	R02 (noncontact)
Application method	Scanning technique (Zig-zag movement)
Pulse energy	500 mJ,^a^ 560 mJ,^b^ 590 mJ,^c^
Pulse width	100 µs
Frequency	10 Hz
Average power	5 W,^a^ 5.6 W,^b^ 5.9 W^c^
Peak power	5000 W,^a^ 5600 W,^b^ 5900 W,^c^
Spot diameter	0.9 mm
Spot area at ceramic surface	0.0064 cm^2^
Average power density	1572 W/cm^2^,^a^ 1760 W/cm^2^,^b^ 1855 W/cm^2 c^
Energy density/fluence	157 J/cm^2^,^a^ 176 J/cm^2^,^b^ 186 J/cm^2 c^

Laser parameters with the same superscript letters are in same application scenarios.

**Table 2:** The removal time (sec) of each thickness group at each laser power parameter.

	1 mm thickness		1.5 mm thickness		Mixed thickness		
Laser Power	Mean±SD (sec)	Median(min-max) (sec)	Mean±SD (sec)	Median(min-max) (sec)	Mean±SD (sec)	Median(min-max) (sec)	*p*
5 W	240±39.69	225(210-285)^a^	900±0	900(900-900)^a^	900±0	900(900-900)^a^	-
5.6 W	180±15	180(165-195)^ab^	900±0	900(900-900)^a^	900±0	900(900-900)^a^	-
5.9 W	160 ± 8.66	165(150-165)^b,x^	350±43.3	375(300-375)^b,xy^	550±43.3	525(525-600)^b,y^	0.021*
*p*	0,035^*^		0.021*		0.021*		

The superscript letters a and b indicates significant difference on columns, x and y on line “5.9 W” laser power. There is no difference with the same superscript letters *p*>0.05. *SD* standard deviation. *Differences are significant.

**Table 3:** Evaluation of dental pulp safety in 1 mm thickness group, which was successfully laser debonded with all tested laser parameters.

Dental Pulp Safety	Laser Power			p*
	5 W	5.6 W	5.9 W	
Safe	3 (100%)^a^	1 (33.3%)^ab^	0^b^	0.043
Unsafe	0	2 (66.7%)	3 (100%)	

*Pearson's chi-square test statistical significance (*p*<0.05). There is no difference with the same superscript letters *p*>0.05.
